# Selections and Abstracts

**Published:** 1905-11

**Authors:** 


### SELECTIONS AND ABSTRACTS.

### Foreign Abstracts.

By Fred G. Hodgson, M.D., Atlanta, Ga.

A Method of Sterilization of Women.—Under the above title there have appeared several articles in the *Monatsschrift fur Geburtshulfe und Gynaekologie* (Berlin). The method first recommended was the excision of a small portion of both Fallopian tubes through the vaginal route. Kustner, of Breslau, however, reported two cases in which this was done, and afterwards both women conceived. He attributes these failures to the fact that the openings into the uterus must have failed to close. He, therefore, recommends a laparotomy and complete removal of the tubes with a wedge-shaped excision of the uterine ends of the tubes and a careful closure by suture of the opening into the uterus closing the peritoneum over this opening, taking great pains to obtain primary union.

Neuman, of Berlin, says the excision of the entire tube is not at all necessary. He makes only a small laparotomy wound, pulls the uterus up into the wound and excises only a small portion of the uterine end of the tube, then cuts out a wedge-shaped piece from each cornua and sutures in the same careful manner so as to obtain primary union. He says this can also be accomplished by the vaginal route, but the suture is more difficult.

The chief indications for this operation are: (1) Marked narrowing of the pelvis; (2) tuberculosis of the lungs; (3) chronic nephritis. Other indications may arise, and it is very easily done during the course of other operations.

The Effect of Salt Upon Nephritis.—The *Semaine Medicale* (Paris) has had several articles recently upon the effect of salt upon diseased kidneys. A number of cases of chronic nephritis with albuminuria and oedema were selected and given a diet absolutely free from salt. It was found that the albuminuria and oedema were markedly reduced or disappeared, when salt was again given with the diet the albuminuria and oedema returned or increased. These experiments were repeated by a number of very competent observers and invariably with the same results. Various explanations were offered, some saying that the salt acted in a mechanical way, causing osmosis; others maintain that the salt is a direct irritant to the kidney cells.

It is thought that the favorable results obtained with an exclusive diet in these cases is due to the fact that milk contains so little salt. Now nephritic patients can be given a much more liberal diet if care is only taken to omit the salt. It was also learned that a number of the patients with nephritis gave a previous history of having been in the habit of eating large amounts of salt. It is, therefore, well to warn all patients against this possible dan ger from an article which is in such universal use.

---

### ALCOHOLIC INSANITY.

Dr. I. A. McSwain, at the Tennessee State Medical Association, April, 1905, offered the following suggestions on this subject: (1) The children of drunken and debauched parents ought, for obvious reasons, to be taken away from them and placed in decent homes, or removed to industrial institutions provided by the State. This would check their hereditary tendencies to drunkenness, and therefore reduce the number being raised up to become a burden to the State in the way of paupers, criminals and lunatics. (2) Young people, who early in life contract the pernicious habit of drinking, should also be removed from the temptation of their environments, and placed in institutions in which they should be taught some useful employment, and restrained from vicious habits. (3) The drinking man, as soon as he began his spree, before he was crazed by it, should be taken into custody, not as a mere nuisance, but as a dangerous man, or one likely to become so, because of insanity in the incipient stage. (4) The confirmed drunkard, the chronic alcoholic subject, should on no account be allowed to exercise his personal liberty in the pursuit of delusions which result from prolonged excesses.

### AN EPITOME OF THE MORE IMPORTANT CHANGES MADE IN THE U. S. PHARMACOPOEIA.

COMPILED FOR THE CONVENIENCE OF PHYSICIANS—JOHN WYETH & BROTHER, INC., MANUFACTURING CHEMISTS, PHILADELPHIA, PA.

### DECREASED STRENGTH.

| NAME OF PREPARATION. | OLD PHARMACOPOEIA OF 1890 | NEW PHARMACOPOEIA OF 1900. |
|---|---|---|
| Syrup Iron Iodide......... | 10% by weight of Ferrous Iodide. | 5% by weight of Ferrous Iodide. |
| Tinct. Aconite............. | 35 gms. to 100 c.c. | 10 gms. to 100 c.c. |
| Tinct. Belladonna Leaves.. | 15 gms. to 100 c.c. | 10 gms. to 100 c.c. |
| Tinct. Cannabis Indica.... | 15 gms. to 100 c.c. | 10 gms. to 100 c.c. |
| Tinct. Colchicum Seed.... | 15 gms. to 100 c.c. | 10 gms. to 100 c.c. |
| Tinct. Digitalis............ | 15 gms. to 100 c.c. | 10 gms. to 100 c.c. |
| Tinct. Gelsemium......... | 15 gms. to 100 c.c. | 10 gms. to 100 c.c. |
| Tinct. Hyoscyamus. ...... | 15 gms. to 100 c.c. | 10 gms. to 100 c.c, |
| Tinct. Iron Chloride....... | Assay, 13.6% by weight of anhydrous Iron Chloride. | Assay, 13.28% by weight of anhydrous Iron Chloride. |
| Tinct. Lobelia. ........... | 20 gms. to 100 c.c. | 10 gms. to 100 c.c. |
| Tinct. Nux Vomica........ | 0.3% total alkaloids (about 0.15% Strychnine Alkaloid). | 0.1% Strychnine Alkal'd |
| Tinct. Opium.............. | 1.3 to 1.5% Cryst. Morphine. | 1.2 to 1.25% Cryst. Morphine. |
| Tinct. Opium, Deodorized.. | 1.3 to 1.5% Cryst. Morphine. | 1.2 to 1.25% Cryst. Morphine. |
| Tinct. Physostigma ....... | 15 gms. to 100 c.c. | 10 gms. to 100 c.c. |
| Tinct. Veratrum Viride... | 40 gms. to 100 c.c. | 10 gms. to 100 c.c. |
| Wine of Colchicum Seed.. | 15 gms. Colchicum Seed in 100 c.c. | 10 c.c. F. E. Colch. Seed in 100 c.c. |

Physicians and druggists should *bear in mind* the change on the potent tinctures, such as Aconite, Belladonna, Didgitalis, Gelsemium, Hyoscyamus, Physostigma and Veratrum. Physicians will be obliged to prescribe larger doses of these tinctures, if the new official strength is dispensed, than when they used and depended on the tincture of the above drugs conforming to the 1890 Pharmacopœia.

### INCREASED STRENGTH.

| NAME OF PREPARATION. | OLD PHARMACOPOEIA OR 1890 | NEW PHARMACOPOEIA OF 1900 |
|---|---|---|
| Acid Sulphuric, Aromatic. | 18.5% by w'ght of Acid Sulphuric. | 20% by weight of Acid Sulphuric. |
| Extract Opium ........... | 18% Cryst. Morphine. | 20% Cryst. Morphine. |
| F. E. Nux Vomica......... | About 0.75% Strych'ne | 1% Strychnine Alkaloid. |
| Sol. Iron and Ammon., Act. | 2 c.c. Tinct. Iron Chlor. to 100 c.c. | 4 c.c. Tinct. Iron Chlor. to 100 c c. |
| Tinct. Cantharides........ | 5 gms. to 100 c.c. | 10 gms. to 100 c.c. |
| Tinct. Capsicum .......... | 5 gms. to 100 c.c. | 10 gms. to 100 c.c. |
| Tinct. Rhubarb ........... | 10 gms. to 100 c.c. | 20 gms. to 100 c.c. |
| Tinct. Strophanthus ...... | 5 gms. to 100 c.c. | 10 gms. to 100 c.c. |
| Tinct. Tolu. ............. | 10 gms. to 100 c.c. | 20 gms. to 100 c.c. |
| Wine of Ergot ........... | 15 gms. Ergot to 100 c.c. | 20 c.c. F. E. Ergot to 100 c.c. |

The physician's attention is especially directed to Tincture Strophanthus. The strength of the tincture as adopted in the 1900 Pharmacopœia is double that of the 1890 edition. Besides these changes a fixed standard of assay has been adopted for a number of drugs, extracts and fluid extracts.

### MODERN THERAPEUTICS AND PHARMACY.*

BY FREDERICK HADRA, M.D., SAN ANTONIO, TEXAS.

In speaking of ethical proprietaries, he says: "I should be sorry, indeed, if the prejudices of any member of this society should so far overcome his better judgment as to banish all or most of these drugs from his practice without investigating their merits. So, if we desire a local antiphlogistic effect, and have to choose between the ancient, unsightly, unhygienic and troublesome flax-seed poultice and the newer proprietary article called antiphlogistine, a physician must needs be prejudiced indeed who will prefer the former . . . It may be a matter of theoretical indifference what preparation we prescribe, but it may be quite a different matter with the patient who has to use it for long periods."

"Does it not strike you as somewhat incongruous that we alone of all professions and trades should rise up in arms against a coordinate branch which is continually striving to assist us in improving our therapeutic weapons? If we would take advantage of the opportunity offered to make intelligent selections of such preparations of drugs of reliable concerns as appeal to reason and common sense, those of us who do so will certainly have an advantage over those who do not."

As regards the refilling by the druggists of prescriptions of proprietary remedies, he says: "If I am called to treat a sprain of the ankle, and find it necessary to order an antiphlogistic application, it would be just as easy for the patient to send to his druggist daily for more flax-seed meal or iodine, as it would be for him to order more cans of the more cleanly proprietary preparation, antiphlogistine. A tonic or cough medicine, quinine mixture or capsule would share the same fate whether proprietary or extemporaneous."

"If the intelligent use of the drugs mentioned is not injurious *per se*, why should we protect the laity against their use any more than against the employment of any other drugs? Would the committee advocate the abandonment of calomel, castor oil, mag. sulph., quinine, flax-seed meal, paregoric, laudanum or carbolic acid because the laity can also go to the drug store and purchase these just as they can cascara preparations, phenacetin, listerine, antiphlogistine, etc.?"

*Extracts from an article in the Texas Medical Journal for March, 1905.

### THE TREATMENT OF EMPYEMATA OF THE MAXILLARY SINUS THROUGH THE NOSE.

Geo. L. Richard (*Jour. A. M. A.*, September 16, 1905) gives the following conclusions:

First determine whether the case is of dental or of nasal origin. If of dental origin, treat it accordingly but do not continue, by tube or otherwise, a long-continued course of treatment through the mouth. If of nasal origin, treat in the manner described. If in doubt, treat through the nose, letting the tooth alone unless it seems to be troublesome. Even this method of treatment do not continue indefinitely if the pus continues to be a source of trouble, but do the radical operation through the canine fossa, a description of which does not come within the province of this paper. Lastly, see whether there is a coexistent empyema of the frontal sinus which may be draining into the antrum and keeping up the discharge of pus.

Professor Gerber of Königsberg* recently published a short but lucid article on the principles of treatment in empyemata of the antrum, which are so in accord with my own experiences in this regard that I wish to translate his conclusions somewhat freely and to incorporate them in this paper. He states that he has tried all the various methods which have been advocated and has finally come to the following conclusions, which will govern him until such time as something better may be brought forward. In dealing with diseased antra of Highmore, the following principles should govern the choice of a method of treatment:

*First.*—Whenever possible, direct the treatment to the point where the antrum has its natural communication with the nose, and especially avoid any long-continued direct communication with the mouth or pharynx.

*Second.*—In cases which are not severe, the antrum is washed out in the beginning through a sharp canula introduced in the middle meatus.

*Third.*—If the empyema has lasted a considerable time and the secretion is thick, with considerable odor, this opening in the mid-

* Archiv. für Laryngologie and Rhinologie, vol. xvii. part I.

dle meatus is next enlarged, the so-called supraturbinal resection of Onodi being performed, to include also the inferior meatus, if it be necessary.

*Fourth.*—In chronic and severe cases it may be necessary to make a large opening from the canine fossa, the so-called radical operation, but this opening, after thorough inspection and cleaning out of the cavity and the making of a broad counter-opening in the middle meatus, is carefully closed and the after-treatment entirely performed through the middle meatus.

*Fifth.*—The only patients who can not properly be treated in this way are those in whom there is a high degree of nasal stenosis rendering this treatment impossible, and those who will neither continue in daily communication with the physician nor learn to apply themselves the after-treatment through the nasal fossa.

---

### TYPHOID FEVER IN RELATION TO THE URBAN AND RURAL POPULATION OF THE UNITED STASES.

Seneca Egbert shows by reference to the United States Census Report on Vital Statistics for 1900, and by means of graphic charts illustrating the numerical data of this report, that typhoid fever is much more prevalent in the rural portions of the country. He attributes this high incidence of the disease to the unsanitary conditions prevalent in the many towns and villages that, being under 8,000 in population, are too small to be classed with the urban and which, therefore, make up a large part of the so-called rural population. He concludes: (1) Popular education as to the causation and dissemination of typhoid fever and similar maladies is especially important in the localities and districts indicated by the charts. (2) It is the duty of physicians to impress upon their typhoid patients the importance and necessity of disinfection measures, not only during but also along after convalescence. (3) The profession should urge improvement, purification and care of public water-supplies, and should induce those depending on private or suspicious sources to protect themselves against the danger of infection. (4) The profession should work for uniform and satisfactory registration laws and methods, as these always reflexly foster improvement in sanitary conditions.—*American Medicine.*

### CHRONIC DIARRHEA.

R. W. Wilcox (*American Medicine*, June 10, 1905) gives the following synopsis: Diarrhea being a symptom, the cause should be ascertained. This may be (1) mechanical; (2) nervous; or (3) hemic. Under each division there are subdivisions, each subdivision representing an etiologic factor, which is the basis for treatment. The last should be considered under (1) dietetic; (2) hygienic; (3) medicinal. Certain remedies and methods should be avoided. Opium is only admissible when the alimentary canal has been thoroughly emptied to check excessive peristalsis. It should be given hypodermically as morphine, in substantial doses, and not repeated. A prescription for opium or any of its preparations or alkaloids should never be entrusted to nervous patients; there is too great danger of habit formation. The tannin preparations are sometimes useful in precisely the same way that they are useful as antidotes in alkaloidal poisoning, by temporarily inhibiting the action of bacteria and their toxins. Bismuth naphtholate, bismuth tribromophenolate, and bismuth tetraiodophenolphthaleinate are more effective. Wilcox emphasizes the importance of recognizing that the diarrhea is not always due to demonstrable changes in the alimentary tract. Much as we dislike to present symptom treatment, yet pending further knowledge we must divide diarrheas into classes, based upon their causation, and then proceed to treat them intelligently.

---

### SICK-ROOM IN WINTER.

In rooms heated by a furnace where there is sickness, Dr. Leroy M. Yale, of New York, advises that the hot air should be made to pass over water to which some Platt's Chlorides has been added, and a towel moistened with Platt's Chlorides kept over the register. When heated by a stove or open grate, a basin containing Platt's Chlorides mixed with ten parts of water should be placed near the fire, and a towel occasionally moistened in this kept suspended in the room.

### THE TREATMENT OF DIARRHOEA IN INFANCY AND CHILDHOOD.

This appeals most strongly to those who are interested in the care of children, especially at the advent of the summer months when the mortality from this malady is more than one-half of those who die under twelve months of age. Two forms of acute diarrhoeas are recognized, one of a simple nature due to the presence in the intestinal tract of some form of food, or a surplus of food, which the child can not digest, the other an infectious diarrhoea in which pathogenic bacteria are present. The dysentery bacillus was isolated by Shiga in Japan in 1898, and following up this announcement, Flexner and his co-workers, at the Rockefeller Institute in 1903, found a bacillus agreeing in all essential points with the Shiga bacillus dysenterrae. These with various streptococci and other pus forming micro-organisms are the pathogenic agent to which the infectious, or " summer diarrhoea " of children is due.

Since only a bacteriological examination will determine at the onset of an acute diarrhoea whether it is of a simple or an infectious nature, such treatment must be inaugurated as will meet either indication.

Much can be accomplished in the way of prophylaxis. Since these troubles are more prevalent in the hot months of the year, and in the more crowded and unsanitary sections of the larger towns, a temporary change of residence to the mountains or the country is to be advocated for those who can avail themselves of such a change. When not available all should be done to make the home surroundings conducive to the child's comfort. The largest, coolest and airiest room should be for the children. The clothing should be only sufficient to protect the body from sudden changes or exposure and to keep it comfortable. All the water given to the infant, and this need not be sparing, should be previously boiled and cooled. If the child is artificially fed the milk and milk foods must be pure and fresh and all vessels and utensils used in dispensing, thoroughly and frequently sterilized.

A beginning diarrhoea should always arouse suspicion, and for twenty-four hours all milk and food should be withheld. Milk is absolutely contra-indicated, and under no circumstances must it be allowed. Plain boiled water may be given if there is much thirst, and when the child is very hungry, albumen water, barley water or whey, may be allowed. If the diarrhoea is simple in nature a mild cathartic, preferably calomel in half-grain doses, followed by castor oil will remove the offending material, and this in all probability will be all the treatment required. Gradually the ordinary regime can be resumed and the child by degrees returned to the breast or milk diet.

The infectious form of diarrhoea occurs more frequently among bottle-fed babies, and ninety-seven per cent. of those who succumb to this form of the disease are fed artificially. Here, as in the simple diarrhoea, the milk either from the breast or cow should be discontinued and for twenty-four hours no food of any character must be given, unless it be albumen water. A baby can be kept for days upon this mixture without danger of reducing the strength. The white of one egg is equal in nutritive value to three ounces of breast milk. A cathartic at the outset is indicated and calomel and castor oil answer every indication. For the vomiting, and this rarely persists after the food is stopped, small doses of calomel, one-twentieth of a grain every fifteen minutes, is usually given. If it still persists, the stomach must be washed out, and usually after one irrigation there is no more trouble. Irrigation of the colon is advisable in all cases, as it hastens the action of the cathartic and removes the irritating and offending material. This should be done twice a day—a saline solution of physiological strength and a fountain syringe with soft rectal tube being preferred. Drugs are of secondary importance when compared with dietetics. Certain drugs that are classed as astringents and antiseptic are frequently used and much vaunted, but their efficacy is disputed. Of these perhaps the most valuable are bismuth and salol, and when combined in a pleasant vehicle may be given with benefit every few hours. When there are indications of collapse and rapid prostration, as in cholera infantum, the injection under the skin of a normal salt solution is indicated in small amounts for the operation is painful.

Too much treatment does more harm than good in these cases.

It is a mistake to be putting too many drugs and foods into an irritable, nauseated stomach. Hours of rest do more good than hours of treatment. No food, fresh air, pure water, and a minimum of medicine are the cardinal points in treatment.—*Southern Medicine and Surgery.*

---

### A NEW LIQUID ANTISEPTIC.

Parke, Davis & Co., have recently introduced a new liquid antiseptic of considerable power, called Cresylone. It contains 50 per cent. of Cresylic Acid and forms clear solutions with water in all proportions.

A two per cent. solution of Cresylone is not only an excellent disinfectant for instruments and hands, but a valuable detergent and lubricant, too. It is said not to injure metallic or rubber instruments, though celluloid articles are apt to become friable under its action.

In the treatment of wounds a one per cent. solution is usually employed, and a two per cent. solution may be used in profoundly septic cases when more vigorous measures are indicated.

Cresylone completely arrests the development of pus organisms, and is, therefore, indicated in the various suppurations with which the general practioner has to contend. In the treatment of otorrhea, irrigation with a one-half per cent. solution is said to be of benefit. A solution of the same strength is of value in the treatment of ozena.

As it removes odor, it may prove of service in gangrene. In cancer of the cervix uteri the application of gauze saturated with a solution of Cresylon removes the odor that accompanies this disease. For disinfecting sputa and stools Cresylone commends itself in the sick-room, hospital ward, schools, prisons, etc.

Therapeutically, the use of Cresylone has been suggested in various pathologic conditions, notably in the treatment of gonorrhea, lupus, tonsolitis, eczema, and cystitis of the female.

### WHO OWNS THE PRESCRIPTION?

One of the daily newspapers in an editorial takes up this question, and before reaching conclusions as to the entitled ownership, reviews the argument set forth by physician, druggist and patient in favor of their claim on the desired order. The physician bases his claim upon the right by creation and right to renew indefinitely, so the paper said; the druggist bases his claim on the right of possession, while the patient claims by right of purchase. The dispute, the newspaper says, is made obscure by treating the prescription as substantial property. The prescription is nothing more than a written order to the druggist, requesting him to put up an article accurately described therein. The patient does not buy the prescription, but the compound called for by the order; consequently the prescription rightfully belongs to the druggist, who retains it as a receipt that the order was correctly filled.—*Med. News, Sept. 23, '05.*

---

### THE WILL OF GENERAL WISTAR.

General Isaac J. Wistar, the founder of the Wistar Institute of Anatomy, an institution affiliated with the University of Pennsylvania, died in Claymont, Delaware, on September 18th. By his will he leaves the residue of his estate to the Wistar Institute. It is estimated that after deducting the private bequests, which amount to about $100,000, the residual estate will be valued at about $650,000. General Wistar directed that his brain be given to the Institute, and that his right arm, which is a good specimen of gunshot ankylosis, be also removed and preserved in the Institute. The remainder of his body is to be cremated and the ashes are to be deposited in an urn in the mural recess provided for it in the Institute.—*N. Y. & Phil. Med. Jour., Sept. 30, '05.*

---

In dealing with infections or injuries of the fingers amputation should be a *denier resort.* This is especially the case with a thumb, the most important of all the fingers.—*American Journal of Surgery.*

### IMPORTANT POINTS IN THE TECHNIQUE OF PERINEAL PROSTATECTOMY.

The following rules are laid down by D. Ochsner (*International Journal of Surgery*, July, 1905): (1) The incision must thoroughly expose the prostate. The horseshoe incision does it more effectually than the unilateral or the median incision, though either of the latter will enable one to enucleate it with the finger or by morcellation. (2) Hemorrhage must be avoided as far as possible. This is best done by keeping in the mid-plane between the urethra and rectum in splitting the septum, grasping the branches of the internal pudic artery on either side before or just after they are cut, and clamping injured veins near the neck of the bladder. (3) The form of retractor used is unimportant so long as it is in a position where it can be kept under control. (4) The traumatism should be reduced to a minimum. (5) If the prostatic urethra is completely removed at any point, the remaining upper and lower segments should be united by catgut sutures through the anterior wall, the posterior wall being left open for drainage. (6) Good drainage is important, no matter how it is accomplished. (7) The duration of the operation should be as brief as possible, in order to minimize shock and the effects of the anesthetic upon the kidneys. The author thinks that by observing these rules the operation will gain in simplicity and lose in gravity.

---

### STARTS A PARASITE EXCHANGE.

Dr. L. O. Howard, who was sent to Europe in June by the Massachusetts entomological officers for the purpose of collecting and shipping to this country parasites for the suppression of the gypsy and brown-tail moths, has returned to Boston. He announced that he had made an agreement with European officers whereby they will ship to this country the insects needed for the moth warfare, in return for American parasites needed abroad. Dr. Howard said that the parasites he had secured merely would keep the moth pest in check, and that propertv owners should not in any way relax watchfulness over their trees.—*Med. News, Sept. 23, '05.*

The Herter Lectures, established by Dr. C. A. Herter, at New York University and Bellevue Hospital Medical College, will be given this year by Professor Carl von Noorden, chief of the City Hospital of Frankfort, Germany. His subject will be Diabetes. The lectures, six in number, will be given in English in the large auditorium of the Carnegie Laboratory, 338 East Twenty-sixth Street, from Monday, October 9th, to Saturday, the 14th, inclusive, at 4 o'clock in the afternoon. Visitors are welcome to these lectures. Reserved seats to be had on application to the college.—*N. Y. & Phil. Med. Jour., Sept. 30, '05.*

---

First Apostle Dowie of Zion City has not only directed that hereafter all members of his church who marry must first have his written consent in order that the union may be recognized by the church, but has also issued a command that in future every couple married increase the population by one child each year. Before marriage, however, even the chaste kiss is forbidden the engaged couple.—*Med. Age, Sept., '05.*

---

### REPORT ON PEACOCK'S BROMIDES.

We have purchased, in London, a bottle of Peacock's Bromides (Syr. Brom. Comp. Peacock) and subjected it to analysis.

We found the preparation to consist of a neutral solution of the mixed bromides of potassium, sodium, calcium, ammonium and lithium.

The amount of bromide present was found to be equivalent to 14.989 bromides in the fluid drachm, thus proving the exactness of the medication in the product.

The salts employed for the solution were particularly free from iodides and bromates, which, of course, is the important factor.

H. HELBING,

F. W. PASSMORE.

London, England.

### IRON IN STOMACH DISEASES.

Elsner, in *Therapieder Gegeuwart*, Berlin, considers it established that iron is absorbed and assimilated, and that organic combinations are the most suitable for therapeutic administration because iron is contained in the food in proteid combination. A difference in the hemogleblin-forming power of the various preparations of iron has not been demonstrated, so that the choice of the various preparations at present should be decided by their effect on the digestive organs. Elsner experimented with a hemoglobin preparation to determine its effect upon the digestive functions. He concludes as follows: Symptoms of dyspepsia do not contraindicate the use of iron preparations, but in such case functional investigation of the stomach should precede the use of iron. Iron preparations are contraindicated in organic stomach diseases, with hyperacidity or hypersecretion, and if existing dyspeptic troubles are increased by the use of iron.—*Journal A. M. A. Sept. 9.*

---

ADENOIDS AND EYE AFFECTIONS.—Hern states that the eyes are often found to be affected in cases of adenoids, the diseases of the eye usually found being: (1) Phlyctenular conjunctivitis (by far the most common). (2) So called weak ulcer of the cornea, the non-inflammatory ulcer which looks as if a small piece of the corneal surface had been gouged out; sometimes difficult to see unless fuchsin is used. (3) Eczematous keratitis, often called phlyctenular keratoconjunctivitis. (4) A peculiar irritability or hypersensitiveness of the retina, leading to difficulty in opening the eyes in a bright light. There can be no reasonable doubt that these ophthalmic conditions are secondary to the nasopharyngeal: (a) By the marked lowering of the general health produced. (b) By the actual extension of the inflammatory process up to the nasal duct to the eye.—*British Medical Journal, August 26, 1905.*

---

When operating for empyema thoracis it is a good rule to aspirate again when the pleura is exposed and before it is incised. This may save some embarrassment.—*American Journal of Surgery.*

### LIQUOZONE VERSUS GLYCOZONE.

Editorial From the Louisville Monthly Journal of Medicine and Surgery, September, 1905.

In a recent issue of the *New England Medical Monthly* is an editorial setting forth the facts that much confusion has arisen over the similarity of name *Liquozone* and *Glycozone*, and furnishes the following incident: "The name 'Liquozone' is confusing druggists and even doctors with another legitimate preparation, 'Glycozone,' to such an extent, that in March last the editor of the *New England Medical Monthly* called at a drug store in Mount Clemens, Mich., asking for a bottle of Glycozone, whenthe clerk turned around and supplied him with a bottle of Liquozone.

"Upon my remark that I did not ask for a bottle of Liquozone but for a bottle of Glycozone, spelling the name at the same time, he apologized, stating that he understood that I wanted a bottle of Liquozone.

"This instance shows plainly that the confusion which has been created not only on account of the fallacious claims, but also on account of the sounding of the two names 'Liquozone' and 'Glycozone,' which is practically the same, is misleading to the limit."

The incident is very similar to one which happened to an editor of this JOURNAL but very recently. He was at Atlantic City, N. J., and sent to a drug store—said to be a first-class one—for a bottle of Marchand's *Glycozone*, when he received a bottle of *Liquozone*. The mistake seemed inexcusable, and goes to confirm what the *Journal* quoted says about the confusion of names. And too, it must be remembered that great injustice is being done an old and tried medicine—Marchand's Glycozone—by a late nostrum put in the field, and which has received the condemnation of the San Francisco Board of Health, in the following words in the report of the Health officer:

"I therefore recommend that it be condemned by the Commissioners of Health as a pernicious and unsafe drug. It may be used as a disinfectant for trains, urinals, stables, etc., and probably no objections could be urged against its external use by an individual to exterminate barber's itch.

"I therefore recommend that the same action be taken in respect to this deleterious drug as is prosecuted by your Honorable Commission in the case of impure food, viz.: that the Police Department and our Department act conjointly to have it removed from the shelves of all dealers handling this drug, and that the selling, exposing for sale or giving away of Liquozone, in the City and County of San Francisco, constitute an offense, the violation of which will be followed by arrest of the offenders.

"Respectfully submitted,

"D. F. RAGAN, M. D.,

"Health Officer."

---

A CASE OF CONGENITAL URETHRAL STRICTURE ASSOCIATED WITH HEMATURIA AND SYMPTOMS SUGGESTING RENAL DISEASE. —J. W. Churchman, (*Johns Hopkins Hosp. Bull.*, July, 1905), after reporting in detail a case of congenital urethral stricture, summarizes the interesting features presented as follows: (1) This patient presented a urethral stricture in which a positive diagnosis of its congenital nature could be made. Such a diagnosis was warranted by: (*a*) The history, from which all record of urethral traumatism or of venereal infection was absent; (*b*) the association of an obvious congenital stricture at the meatus; (*c*) the complete cure of the clinical manifestations by proper treatment of the stricture; (*d*) the presence of clear urine and the absence of symptoms after the actual stricture had been dilated. (2) The second point of interest was offered by the clinical symptoms presented. These were nausea and vomiting, hematuria and pain over the left ureter. They suggested renal or urethral disease, and obscured the diagnosis which really only cleared up by the therapeutic test. (3) In the third place, this case suggests that another item must be added to our already long list of causes for hematuria, and that congenital stricture may be associated with blood in the urine, the source of which may probably be an hypertrophied veru montanum.

---

In excising a varicocele under local anesthesia, tie the upper ligature first; the pain of tying the lower ligature will then be abolished.—*American Journal of Surgery.*

### INTUSSUSCEPTION IN INFANCY AND CHILDHOOD WITH COLLECTION OF 1,028 CASES, WITH STATISTICS.

J. H. Hess, in Archives of Pediatrics, September 1905, gives the following summary of the treatment:

*Summary of Treatment.*—(1) Intussusception demands an early diagnosis and immediate treatment.

(2) Abstinence from all food: far more important, purgation must absolutely be prohibited. The question of sedatives in the form of opium, etc., must rest with the physician.

(3) Irrigation may be tried once or twice under the proper condition and in properly selected cases.

Conditions:—

(*a*) Preparation for immediate laparotomy in case of failure.

(*b*) Complete anesthesia.

(*c*) Hot salt solution or plain water may be used under a pressure of not more than three feet, the fluid being allowed to remain in the bowel not less than ten minutes.

(4) Contraindications to irrigation.

(*a*) Recurrence after a previous complete or partial reduction.

(*b*) The very acute and severe types of this disease, which result in early destruction of the bowel wall, but which cases are fortunately not the most frequent type.

(*c*) Where there are signs of beginning gangrene or ulceration, evidenced by subnormal temperature, profound toxemia, and other septic symptoms.

(*d*) Enteric intussusceptions.

(5) Laparotomy should follow failure of irrigation without delay.

(*a*) Attempted simple reduction from below upward.

(*b*) In irreducible cases. Resection within the bowel in selected cases, or, where this is not feasible, resection with end to end anastomosis should be attempted where the patient's condition makes it practicable, as an artificial anus or simple packing about the bowel requires a secondary, and only too frequently fatal, operation.

---

In typhoid fever spontaneous rupture of the spleen may simulate intestinal perforation.—*American Journal of Surgery.*

ACIDURIA FOLLOWING CHLOROFORM.—Guthrie's conclusions are: (1) That both ether and chloroform are dangerous under certain condition at present undetermined. (2) That the symptoms suggest acid intoxication by the poisonous precursors of acetone. (3) That the origin of such poisonous bodies (-oxybutyric acid, etc.) is in the disintegration of fat. (4) That in practically all cases of death following anæsthetics, advanced fatty metamorphosis is found in most organs, and in the liver especially. (5) That acid intoxication arises from such fatty metamorphosis in organs. (6) Although prolonged administration of chloroform certainly produces general fatty metamorphosis, it is incredible that such profound changes can be induced by small doses given during short operations. (7) Ether is not capable of producing similar changes, yet they are found apparently to the same extent in deaths following ether as in deaths after chloroform. (8) It therefore follows that such fatty metamorphosis must have existed prior to the anæsthetization. (9) The disintegration of fat into acid poisons may be due to the direct action of chloroform and ether in altering normal metabolism, or in some way they may favor the action of bacterial toxines present in the intestines in causing disintegration of fat. (10) In any case the preexistence of advanced fatty changes must be presumed in order to explain fatty acid intoxication. (11) It is probable that the fatty changes in the liver are physiological and of the nature of infiltration rather than of degeneration. (12) This would explain why anæsthetics are dangerous at one time, and not at another, the element of danger being the superabundance of fat existing in the liver at the time of operation. (13) The storage of superfluous fat in the liver may in some cases be due to the large quantities of cod liver oil and fatty diet commonly supplied to delicate, crippled, and rickety children. In accordance with the above given views the following precautions should be adopted: 1. Before operating on even fat and apparently healthy children, inquiry should be made as to the previous occurrence of so-called "bilious" attacks which may in reality be those of "acidosis." 2. In all cases where over-fattening and want of exercise are suspected, operation should be delayed until the patient has been for some days on fat free diet. Mild purgation is also indicated. The urine should be examined for diacetic acid; if it be present a course of alkalies, such as bicarbonate of sodium, should be prescribed. 3. It should be remembered that both starvation and fright give rise to acetonuria. Instead of a four hours' fast before operation nutrient enemata should be given. Fright can not be altogether controlled, but may be combated by preventing starvation. 4. The treatment of symptoms of acid intoxication following operations should be by venesection, saline transfusion, and by clysters of solution of bicarbonate of sodium.—*Lancet, August 26, 1905.*

---

F. P. Kimmicutt (*Medical Record*, June 10, 1905) in a "contribution to Hemophilia" gives the following summary: (1) The internal administration of the lime salts, especially of the chloride of calcium, in doses of two grammes (30 grains) twice or thrice daily. Its continued use should be interrupted every two or three days by a period of twenty-four hours. (2) The application of solutions of the same salt with compression, for accessible surface bleedings. Aqueous solutions of one-half per cent. are efficient. Another convenient mode of employment is in the form of a finely powdered chalk mixed with one-half per cent. solution of the calcium chloride. The less soluble lime salts, such as calcium phosphate, are equally efficient for local application. (3) The use of a combination of one-half per cent. solution of calcium chloride and one-sixth volume of a solution of nucleoalbumin for local application. The nucleoalbumin can be obtained from aqueous extracts of various cellular tissues—testicle, thymus, thyroid, ovary, etc. If the prepared extracts are not available, a supply may be procured readily by mincing any of these tissues in a weak alkaline solution (1:500 solution carbonate of sodium), and filtering through gauze after a few minutes. (4) The application of a stream of carbonic acid gas to the bleeding surface, in accessible hemorrhage; its inhalation, freely mixed with ordinary air or oxygen, in concealed hemorrhage. (5) The use of four-per-cent. solution of chloral hydrate for surface bleeding, and also of adrenalin (1:1000), with compression.

### REPORT OF DR. CH. WARDELL STILES, GOVERNMENT ZOOLOGIST AT WASHINGTON, ON THE ANTHELMINTIC TREATMENT OF HOOKWORM DISEASE.

"The day before treatment the patient is placed on a milk and soup diet for three days.

"THYMOL: The directions usually given for thymol treatment are: Two grams (31 grains) of thymol at 8 a. m.; 2 grams (31 grains) at 10 a. m.; castor oil or magnesia at 12 noon.

"One week later the stools should be examined, and if the eggs are still present, treatment should be repeated until the eggs disappear, but it is not best to give the thymol more than one day per week. Some cases of hookworm disease are quite obstinate and require a treatment extending over several weeks. It is, therefore, an unfortunate error to expel a few worms with one or two doses and then dismiss the patient as cured without having made further microscopic examination for eggs. * * * * * *

"The administration of thymol has for its object the expulsion of the parasite, hence the removal of the cause of the disease. This should be supplemented by efforts to build up the depleted system by means of good nourishing food, iron, etc. It is well to give the iron daily, except on the days that thymol is taken. * *

"Blotter Paper Test.—For persons who are not in a position to make a microscopic examination, a blotting paper test will be found very useful. Use only fresh feces. Place an ounce or more of the stool on a piece of white blotting paper; allow it to stand for twenty to sixty minutes; remove the feces and examine the color of the stain. In four out of five cases of medium or severe uncinariasis, the stain is reddish-brown and reminds one of a blood stain. In making this test on anæmic patients, piles, of course, should be excluded."

---

GENTLEMEN: We have just issued the 7th of our series of twelve illustrations of the Intestinal Parasites, and we will send them free to the physicians on application.

Yours very truly,

BATTLE & Co

### GUN–SHOT FRACTURES OF THE SKULL IN THE RUSSO–JAPANESE WAR.

M. I. Glagolyev in Chirurgia (Moscow and St. Petersburg) reports ten selected cases, and from his observations concludes:

1. In gun-shot "contact fractures," of the skull it is difficult to determine the extent of injury by ordinary means (inspection, palpation and probing).

2. When a bullet acts on the skull by contact there may be a fracture of the external plate only or of both plates.

3. Between the depressed external plate and the uninjured internal plate foreign bodies may at times be found.

4. In gun-shot "contact fractures" of the skull, we may find torn dura and destroyed portions of brain substance without the presence of abnormal manifestations either psychical, motor or sensory. On the other hand we may have cases in which even the dura is uninjured and yet manifestations of focal injury to the brain are present.

5. In some cases with suppuration of the wounds of the soft parts we find suppuration of brain wound also, but this is determined only by operation.

6. Suppurating wounds of the brain may not cause any rise in temperature even if the pus is under pressure.

7. It is difficult to decide before operation whether dura or brain substance is injured and whether there is suppuration of the brain substance or not.

8. From the above Glagolyev deduces that in order to insure rapid recovery and prevent complications, such as meningitis and abscess of the brain, operative measures are indicated.

9. Conservative treatment of infected gun-shot fractures of the skull is fraught with dangers to the patient's life. In support of this Glagolyev reports a case thus treated.

---

In an acute condition simulating intestinal obstruction, if a large mass can be felt in the abdomen think of omental torsion.—*American Journal of Surgery.*

### THE MOSQUITO AS AN ETHNOLOGICAL FEATURE IN DISEASE.*

BY D. MUNROE, M.D., CAMERON, TEXAS.

Malaria and yellow fever are by far the most prevalent diseases of our tropical country, and plays an important part in the sickness of our Southern States, while yellow fever only occasionally intrudes upon us, from our Southern neighbors, devastating our fair Southland, depopulating its cities and scattering death and destruction in its path.

Malaria stalks close to us, like a witch's fiend, ever ready to do us harm, but by constant association and frequent visitation we become accustomed to its menaces and heedless of its dangers. Although, in a cycle of years it claims more victims than the dreaded yellow fever, which always creates a panic, because of its infectiousness, infrequently the high death rate, besides in its presence we always feel our helplessness, while not so with malarial fever, for we have faith in a specific, and with it combat the disease, regardless of the primary cause.

Until about twenty years ago the medical world universally believed that this something we call malaria' was caused by a miasm, arising from the decomposition of dead vegetable matter, in the presence of both heat and moisture, in a warm climate, and the breathing of this noxious miasm produced malarial diseases in all their varied forms from simply a headache due to malarial toxemia to the pernicious variety, according to the virulency of this deleterious gas, the nature of which we knew nothing.

Now, malarial fever and yellow fever were thought to be kindred diseases, because of the similarity of their symptomatology and etiology, both being indigenous to the same country and both being influenced by the same environments, as well as increased in malignancy by the same unsanitary conditions. Hence, we should naturally expect their etiological factors to be found along similar lines by the scientific investigator.

Even the wind was thought to be an important element in the dissemination of each of these diseases, because we believed it wafted these noxious causes over the country. The longer the wind blew,

---

*Read before the Milam County Medical Society.

the greater would be the distance traversed by this deleterious something, and greater the area covered by it. Hence, the more people afflicted by these diseases after a steady wind from a particular direction. For this reason, during the summer, the folks on the south side of the streams in Texas dreaded a northerly wind, while those on the north side, for a like reason, feared a southerly wind. Each party recognized the fact that such winds forbode evil to them.

I have seen almost the whole family in bed, suffering from some form of malarial toxemia, which was attributed by them to the direction of the wind, and which was acquiesced in by the family physician.

On the south side of Little River, in Milam county, is a lake covering about forty acres of land, within a short distance of which are several residences, in different directions from this body of water, and those living therein know they are almost certain to suffer more or less from malarial sickness if the wind blows a few days over this lake towards their dwellings. In one family, near this lake, were two cases of the pernicious form of malarial fever at different times, following a north or northwest wind. In fact, the family had been living there in the same place for twenty years, and noticed they always had more or less sickness after such winds.

I advised a man to drain a tank, which was east of his house and near by, for the reason the prevailing winds during our hot summers come from the east or southeast and blow this materies morbi, or miasm, from this tank over and through his dwelling, and thus infect his family with malaria. He heeded my advice, and as a consequence escaped his usual endemic of malarial troubles the following summer.

Now, I yet believe, the near-by water and the wind played a very potent factor in the production of this malarial sickness, both being rather indispensable means, the water, however, being the incubator and not the generator, as was formally supposed, the wind playing the part of a disseminator.

Among the possessions wrested from Spain as trophies of the war, was Cuba, the acknowledged home of yellow fever; while Havana, the capital city, was regarded as the nidus for yellew fever germs for the whole world because of its unsanitary condition, and had, for that reason, been considered a constant menace to our Southern border for over a century. So, when the United States army occupied Cuba, and took charge of this infected city, an army physician was placed at the head of its health department, and was allowed a free hand and unlimited means to do what he thought requisite to free the city of yellow fever. Accordingly, he set his force to work to clean this foul, dirty and filthy city, as Ben Butler did New Orleans in 1862. So Havana soon became a clean city, and in a comparatively good sanitary condition by means of scavenger wagons, incinerating stations, and police regulations, together with a modern system of sewerage. This, with strict isolation and efficient quarantine, constituted their sole efforts to rid the city of yellow fever. Notwithstanding such drastic measures were employed to place the city in a clean and sanitary condition, yellow fever continued in its work of sorrow and death, while malarial fever showed but little signs of abating.

Many prominent American citizens, both civil and military, succumbed to both diseases during the year 1900, and that, too, in the very cleanest and most sanitary portion of the city, and among those people who lived the best and took the best care of themselves. The Surgeon-General of the United States army, fully appreciating the thoroughness of the work done by the department of health during the year 1900, as well as its inadequateness to control the fever situation of the city, in 1901 dispatched Havana a new board of army medical men for the express purpose of investigating, specifically and definitely, the cause of yellow fever. When this body of scientific physicians arrived in the infected city, seeing the futile efforts of the board of health to stamp out the disease by their work, so carefully, so thoroughly, and so efficiently done, they resolved to abandon the beaten paths of etiology and investigate along new lines of thought, and after mature deliberation, much parleying and some investigation and reasoning from analogy as well, they agreed to study the relation of the mosquito to yellow fever, for the following reasons:

First, the city of Havana and Los Vegas, a suburb of the city, were swarming with these little pests, and the air very resonant with their hums.

Second, Dr. Carlos Finlay, a prominent physician of Havana, believed, from certain experiments which he had made, that the mosquito had something to do with the spread of yellow fever, but he had not been able to verify it for the lack of police authority and the want of means.

Third, that it was claimed by Dr. Roland Ross, an eminent English army surgeon of India, that the mosquito played some part in the production of malarial fever.

All of which served as beacon lights to this board of doctors in their search for the cause of yellow fever. Now, for their investigations to be convincing and conclusive, they had to be very careful and precise in the details, as well as very exact in the whole plan of their investigation. Now, these medical men did a great deal of experimenting along different lines before they arrived at the following conclusion: That if a female mosquito, the stegomyia variety, was permitted to bite a yellow fever patient during the first three days of his sickness, and then allowed to live twelve or fifteen days thereafter, she then became an infected mosquito and capable of producing a typical case of yellow fever. To prove the correctness of this conclusion, they selected a plot of ground as an experiment station about six miles out from the city of Havana, around which was stationed a military guard, to interdict any communication with the city or adjacent country, where a small frame building was erected and securely screened so as to prevent the egress or ingress of mosquitoes to this house. The eggs of the stegomyia variety was put in breeding jars containing water, where they were allowed to hatch and develop into a winged mosquito. Now, the male mosquito is distinguishable by his long antennæ extending out from his head; he was rejected for experimental purposes, as he will not bite. Therefore, a female mosquito of this variety was selected and carried to Havana in a glass jar with a cotton stopper, and allowed to bite a patient suffering from yellow fever during the first three days of his sickness; and after filling herself with this contaminated blood, she was rebottled and returned to the same room from which she was taken and put in a glass jar again in which was placed some white sugar and water, upon which she could feed and live, covering the mouth of the bottle with mosquito netting, where she was permitted to remain and digest this contaminated blood for twelve or fifteen days. Now, at the expiration of this time they had an infected mosquito ready for experimental purposes, as per former decision.

At this experiment station they also constructed a large room and securely screened it with wire netting, the meshes of which were not larger than sixteen to the inch, and partitioned it into two rooms with the same material. Now, into each of these rooms were put one volunteer, heroes of science, who allowed themselves to be experimented on for the benefit of science and the good of humanity, where these men remained perfectly well for two weeks. Then one of these same men permitted one of these infected mospuitoes to bite him, and in five days this man had yellow fever, while the man in the other room, separated only by wire netting, breathing the same air and with the same environments and subjected to the same conditions, remained well and free from any symptoms of yellow fever. Next they emptied the room where the man contracted yellow fever and placed therein a bed and furnished it with mattress, sheets and pillows soiled with the feces and black vomit of a yellow fever patient, and admitted therein a volunteer, who occupied this room, slept on this soiled mattress between these soiled sheets for two weeks, and escaped yellow fever so long as he was kept safe from the bites of an infected mosquito. These discoveries rapidly changed the duties and labors of the health department of this infected city of Cuba, for a new light dawned upon the scene. Consequently, in 1901, the Board of Health of Havana, Cuba, raised the black flag, and waged such a relentless war on the whole mosquito family, present and prospective, and continued the fight in and around the city for three years, until these pests were almost exterminated in the resident portion of the city by both killing the mosquitoes in the houses and destroying their adjacent breeding places. The cessation of yellow fever and malarial fever were coincident with the disappearance of mosquitoes from that locality.

While in 1900 the yellow fever was very severe in and around

Havana, and 325 deaths were reported from malarial fever alone, during 1901, the year the mosquito war began, yellow fever diminished some, while deaths by malarial fever decreased by more than half; while in 1902 the same ratio of decrease was reported in both diseases. Until in the year 1903 there was scarcely any yellow fever in this Cuban city, and only forty-five deaths reported from malarial fever.

There is no longer a question of doubt but that yellow fever is transmitted by inoculation, and that the female mosquito, of a particular species, does the inoculating.

Dr. Roland Ross, an investigator and an English army surgeon of India, with the microscope found the malarial parasite in certain species of mosquitoes he examined in that country, and traced these parasites, by the aid of the microscope, into the salivary glands of this same mosquito, where they were found in great numbers.

It is an acknowledged fact, when the mosquito bites she injects into the wound her saliva, and this saliva causes the irritation, swelling and pain of an ordinary mosquito bite.

Now, Dr. Ross reasoned that this mosquito injected these malarial parasites along with her saliva into the wound, and thereby inoculated the person so bit with malaria. To prove this to be a fact, he allowed a female mosquito to bite a patient suffering with malaria toxemia, and then transmitted her to London, England, where malaria is unknown, and allowed this same mosquito to bite a young man who was free from malarial poison, and in five days he was stricken down with malarial fever, in whose blood was found the malarial parasite by the microscope. Furthermore, some English investigators spent quite a while in Campania, Italy, a place recognized as intensely malarial, living in a mosquito-proof house, made so by carefully and securely screening the entire house, so as to effectually keep out all mosquitoes. In this house they remained free from all malarial troubles, and when they departed there was no malarial parasites found in their blood by the microscope, which fact, beyond a shadow of a doubt, proves that they were free from malarial poisoning.

There is another mosquito-bearing disease called filariasis, found in tropical countries, especially in Africa and the East Indies, among our wards—the Filipinos. Dr. Manson, an eminent English surgeon, in his investigations and researches, decided this parasite disease was communicated to man by the bite of a certain mosquito of those countries. This parasite gives rise to an intense inflammation, subsequent swelling, and resulting in an abscess, with its accompanying sequelæ.

It is no longer a debatable question, but a demonstrated certainty, that malaria, in all its varied forms, is transmitted to man solely by the bite of a certain species of mosquito found in the so-called malarial localities, while it is also a proven fact that a tropical member of the mosquito family inoculates the human race with the yellow fever germs. Therefore, the mosquito is no longer a pest to be endured, but is a serious menace to health at all times, and for that reason should be exterminated. To do this necessitates our studying the life and habits of the different species of mosquitoes found in our own Southern country.

Both the malarial and yellow fever mosquitoes breed and live near, or in the house, and do not wander over 600 yards from their breeding-place. The female lays her eggs in still, fresh water, and they hatch out in two or three days into an air-breathing wiggler, and during the five days in which they remain in this stage they are compelled to come to the surface of this water every few moments to get air to breathe, and at the expiration of this time they develop into the characteristic winged mosquito and fly about the premises, hunting whom they may bite and annoy and inoculate.

The mosquito is full grown in a day, and lays eggs the third day. An ordinary tub, wash-pot, or barrel, containing water, will hatch 200 mosquitoes per day, and a few bottles, tin cans, buckets, puddles, troughs, rain barrels, cisterns or cesspools scattered around the premises, will supply an innumerable number of mosquitoes to keep the residents sick and unhappy all summer.

But if each householder will see to it that every receptacle holding water on or near the premises are emptied, drained, oiled or securely screened, and attend to this duty every week during the warm weather, he will raise no mosquitoes on his premises to annoy and infect himself and family nor that of his neighbors.

To free his house of all mosquitoes, each room should be securely closed by stopping all crevices and cracks, and make therein a smudge of either sulphur, tobacco, or powdered pyretheum, or even formaldehyde, and keep the room thus closed for three hours, at which time all the mosquitoes in the room will be dead. Then carefully screen the house, and the inmates of that house are safe from all mosquito-bearing diseases so long as they are diligent about their duties around and about their premises.

It is such an easy and simple matter to destroy the breeding places of mosquitoes within the resident portion of our town, and a few words of instruction will usually be sufficient to enlist the attention and induce householders to remove or destroy these objectionable conditions necessary for the breeding of mosquitoes.

The town council should pay strict attention to the proper drainage of all streets and alleys, all public and vacant lots, as well; they should have all low places filled with earth or drained. They should also attend to the draining of all puddles and pools found in the ditches along the streets and alleys. If they would devote their attention to these matters and spend the street money in this work, instead of cutting the weeds down to rot and obstruct the ditches, and thus form pools along these waterways to breed mosquitoes, we would have a healthier town.

When it is impracticable to drain the water off or fill in the low places with dirt, then the surface of the water should be covered well with oil, a gallon of which will cover 2,000 square feet of water surface, which makes it a cheap as well as an excellent temporary remedy. This, to be an effective remedy, must be done every two weeks.

While the town council is legislating against such nuisances as privy pits, hog pens, pigsties, feed pens and slaughtering lots, they should also legislate the wiggler out of existence, for while all of these are very obnoxious, yet, two mosquitoes are more annoying, and by far more dangerous to health, than any of them.

If each citizen of our town would do his whole duty in this matter relative to his own premises, and the city authorities would attend to their duties in reference to drainage, filling in or covering with oil all places containing water, we would not have any malarial sickness in the town of Cameron.

---

Guy C. Kinnaman (*Journal A. M. A.*, August 26th and September 2d), after a long series of painstaking experiments, reaches the following conclusions concerning the antiseptic power of iodin:

1. In a solution of iodin, varying from a 0.2 per cent. to a 1 per cent., we have a germicidal agent of very marked potency.

2. Its germicidal power is far superior to that of bichlorid of mercury, the acknowledged leader of all other antiseptics. This fact was shown by experiments made with a 1/1000 solution of bichlorid of mercury on *Streptococcus pyogenes*, using the same method employed with iodin solution. It was found that an exposure of fifteen minutes, although showing considerable inhibitory power, especially on the first day, permitted a good growth of streptococci to appear. An exposure of thirty minutes gave no growth. The superiority of iodin is readily evidenced by recalling the fact that a comparatively weak solution of iodin, i. e., 0.2 per cent., gave death after two minutes' exposure.

3. It approaches nearly to the ideal antiseptic in that:

(a) It is easily prepared and is stable.

(b) It is non-toxic and non-irritating in strength effective, being only one-fourth as toxic as bichlorid of mercury.

(c) It does not coagulate albumin or form inert compounds with tissues.

(d) It is effective in a very brief time.

(e) The stain it produces soon disappears.

(f) Lastly, and most important, it possesses a remarkable penetrating power.

4. In conclusion, it is well to state that an 0.5 per cent. solution is amply strong enough for all practical usage.

---

The painfulness of withdrawing packings that have dried in a wound may be avoided by soaking them with peroxide of hydrogen.—*American Journal of Surgery.*

### URINE, PRESERVATION OF.

Boracic acid is the most practical urinary preservative that we possess when used in the proportion of five grains to four ounces (or two and one-half grains to two ounces) of urine. Formaldehyde should be used only by the physician or a responsible person. It should be remembered that one drop of the solution will preserve a pint of urine for about a week, and that one drop can be used in four ounces of urine without harm. Other substances than boracic acid and formaldehyde should not be used. The name of the preservative and the quantity that has been used should always accompany the specimen to be examined.—*J. B. Ogden* (*Boston Medical and Surgical Journal, June 22, 1905*).

---

### THE STAGE OF EXHAUSTION.

In the treatment of alcoholism and dipsomania, the physician is called to the case at the stage of exhaustion or prostration and a general derangement of nearly every function. Neurosis, cerebral congestion, cardiac acceleration, gastric and mesentric disturbance, nausea, retching, intolerance of food, intense irritation, insomnia and an endless variety of morbid sequelae, require prompt attention.

It will be found that antikamnia in combination with codeia will give a most prompt and satisfactory response in relieving all the array of symptoms so distressing and usually so obstinate as to defy all ordinary therapeutical interference. The best method is to administer one Antikamnia & Codeine Tablet (antikamnia gr. 4¾, codeine gr. ¼) every fifteen minutes to a half hour, until three are taken, then widen the interval to one and a half to two hours, according to the urgency of the symptoms. Under this treatment the circulation will modify, the cardiac pains subside, the tremor, anxiety and morbid vigilance will give way to rest, quiet, calm and peaceful sleep. The nausea and vomiting, together with the irritable coughs which so frequently characterize these cases, will all disappear.

The superior results obtained with "Antikamnia & Codeine Tablets" are due, in a great measure, to the fact that the manufacturers refine and purify all of the codeia which enters into these tablets, and this prevents the constipation, depression and habit which frequently follow the administration of preparations containing ordinary commercial codeia.

